# Exosomes Transmit Viral Genetic Information and Immune Signals may cause Immunosuppression and Immune Tolerance in ALV-J Infected HD11 cells

**DOI:** 10.7150/ijbs.35839

**Published:** 2020-01-22

**Authors:** Fei Ye, Yan Wang, Qijian He, Can Cui, Heling Yu, Yuxiang Lu, Shiliang Zhu, Hengyong Xu, Xiaoling Zhao, Huadong Yin, Diyan Li, Hua Li, Qing Zhu

**Affiliations:** 1 Institute of Animal Genetics and Breeding, Sichuan Agricultural University, Sichuan, Chengdu, China; 2 Guangdong Provincial Key Laboratory of Animal Molecular Design and Precise Breeding, Foshan University, Foshan, 528231, Guangdong, China

**Keywords:** macrophage cells, retrovirus, exosome, proteome, transcriptome, immune response

## Abstract

Avian leukosis virus (ALV) is oncogenic retrovirus that not only causes immunosuppression but also enhances the host's susceptibility to secondary infection. Exosomes play vital role in the signal transduction cascades that occur in response to viral infection. We want to explore the function of exosomes in the spread of ALV and the body's subsequent immunological response. RNA-sequencing and the isobaric tags for relative and absolute quantitation (iTRAQ) method were used to detect differentially expressed genes (DEGs) and differentially expressed proteins (DEPs) in exosomes secreted by macrophage cells in response to injection with ALV subgroup J (ALV-J). RNA-sequencing identified 513 DEGs in infected cells, with specific differential regulation in mRNA involved in tight junction signaling, TNF signaling, salmonella infection response, and immune response, among other important cellular processes. Differential regulation was observed in 843 lncRNAs, with particular enrichment in those lncRNA targets involved in Rap1 signaling, HTLV-I infection, tight junction signaling, and other signaling pathways. A total of 50 DEPs were identified in the infected cells by iTRAQ. The proteins enriched are involved in immune response, antigen processing, the formation of both MHC protein and myosin complexes, and transport. Combined analysis of the transcriptome and proteome revealed that there were 337 correlations between RNA and protein enrichment, five of which were significant. Pathways that were enriched on both the RNA and protein levels were involved in pathways in cancer, PI3K-Akt signaling pathway, Endocytosis, Epstein-Barr virus infection. These data show that exosomes are transmitters of intercellular signaling in response to viral infection. Exosomes can carry both viral nucleic acids and proteins, making it possible for exosomes to be involved in the viral infection of other cells and the transmission of immune signals between cells. Our sequencing results confirme previous studies on exosomes and further find exosomes may cause immunosuppression and immune tolerance.

## Introduction

Cells continuously secrete different types of micro vesicles into the extracellular space in response to cellular stimuli. Exosomes are membrane-bound micro vesicles that are 40-100 nm in diameter and originate from endocytic compartments within the cell 1. Exosomes are secreted from several different cell types, such as epithelial cells, macrophages, dendritic cells, and frequently contain fragments of mRNA and DNA 2. Secretion of exosomes is thought to be related to the growth and reproduction of many primary and metastatic tumors 3-6, and may be potential biomarkers for disease 7, 8.

Avian leukosis virus (ALV) has become an epidemic, with severe outbreaks occurring in chickens in China and resulting in tremendous economic losses to the poultry industry. The ALV subgroup J (ALV-J) is a human immunodeficiency virus (HIV)-like oncogenic retrovirus that causes immunosuppression. It is thought that this is possibly due to changes that occur in B and T cells upon infection 9, but the mechanism is still unclear. Recently, studies have begun to look into exosomes and the roles they play in immune response as possible explanations for the immunosuppression caused by ALV-J infection. Curiously, chicken biliary exosomes have been observed to promote the proliferation of CD4^+^ and CD8^+^ T cells and hepatic monocytes, which can result in the inhibition of ALV-J infection 10. However, further research has indicated that this effect is dose-dependent. Studies have found that low doses of exosomes isolated from ALV-J infected DF-1 cells activate the immune activity of splenocytes, whereas high doses induce immunosuppression in these cells which suggest that exosomes supply ALV-J with a microenvironment conducive to viral replication and transformation 11. Additional research will further clarify the dose-dependent effect of exosomes on ALV-J infection.

Current the research of transcriptome and proteomics is more about the RNA and protein in cells or immune organs injected by ALV-J to figure out the pathogenic mechanism for the diseases 12. According to the RNA-seq technology, Lan combined four datasets: ALV gene expression, lncRNA, microRNA and mRNA to reveal pathology and inflammatory response mechanisms and the combined analysis revealed that the defenses gene family plays an important role in the immunosuppression caused by ALV-J 13. The change of ALV-J injected DF-1 cells proteomic was detected by iTRAQ, and METAP2, PRDX1, ACTR3, ACTR5 may play vital roles in mechanism of ALV-J injection 14. However, fewer studies have focused on the relationship of exosomes with ALV-J, what's more less the relationship between exosome proteins and RNA.

To explore the function of exosomes in the spread of ALV and the body's subsequent immunological response, in our study, we used HD11 cells, which is a macrophage like immortalized cell line, that derived from chicken bone marrow and transformed with the avain myelocytomatosis type MC29 virus 15. To obtain insight into the expression patterns of mRNA, lncRNA and protein of exosomes, we characterized the RNA transcriptome by high-throughput RNA sequencing and the protein by iTRAQ at seventh day of exosome of ALV-J infection in HD11.

## Materials and methods

### Cell culture

HD11 cells were kindly provided by Prof. Guobin Chang (Department of Animal Science, University of Yangzhou, China). HD11 cells were maintained in a 5% CO_2_ humidified incubator at 37°C. RPMI 1640 media (Gibco) supplemented with 10% heated-inactivated fetal bovine serum (FBS) was used to maintain the HD11 cells. Approximately 5.0 x 10^5^ HD11 cells were cultured in 75 cm^2^ culture flasks for 24 h and then treated (VT) with NX0101, a strain of ALV-J. Uninfected cells (NC) were used as a control. There were *n*=3 replicates.

### Exosomal purification

Exosomes were extracted from the supernatant of HD11 cell cultures using the ExoQuick-TC kit (SBI) with some modifications to protocol 16. Cells were harvested seventh day post-infection with ALV-J. The supernatant was centrifuged at 17,000 x *g* for 10 min at 37°C and the supernatant (S1) was transferred to a new, sterile vessel. The pellet was dissolved in 500 μl of isolation solution (250 mM sucrose, 10 mM triethanolamine, pH 7.6) and incubated in 100 mg of DL-dithiothreitol (DTT) for 10 min at 37°C. The solution was centrifuged at 17,000 x *g* for 10 min at 37°C and the supernatant (S2) was collected. S2 was combined with S1, and 3.3 mL of ExoQuick-TC solution was added before mixing by tube inversion. The mixture was stored for 12 h at 4°C and then centrifuged at 10,000 x *g* for 30 min at 25°C to yield the exosomal fraction (pellet). The exosomal fraction was resuspended in 200 μl of sterile 1X phosphate buffered saline (PBS).

### RNA-sequencing

The total RNA of the exosomes was extracted using TRIzol reagent (Takara, Japan) and eluted in 10 μl of RNase-free water, according to the manufacturer's instructions 17. CDNA libraries were generated according to standard procedure for sequencing analysis. The Ribo-Zero rRNA Removal Kit (Epicentre, USA) was used to remove rRNA from total RNA according to the manufacturer's instructions. The rRNA-depleted RNA was fragmented. The RNA was then reverse transcribed into cDNA using the TruSeq Stranded kit (Illumina, USA) according to the manufacturer's protocol. The libraries were sequenced using the HiSeq 2500 platform, employing paired-end sequencing (BGI, Shenzhen, Guangdong Province, and China).

The raw reads were filtered using Short Oligonucleotide Analysis Package (SOAP) software to remove reads of low-quality, reads containing adapter sequences, and reads containing poly-N sequences 18. After filtering, all clean reads were aligned using Hierarchical Indexing for Spliced Alignment of Transcripts (HISAT) software and assembled using StringTie software. Each transcript had a fragments per kilobase of transcript per million mapped reads (FPKM) value of greater than or equal to zero, with a read coverage greater than one and a length greater than 200 nucleotides (nt). The assembled transcripts were annotated and grouped into different categories using the cuffcompare program from the Cufflinks package 19 on the NONCODE database 20.

LncRNA and mRNA were separated by CPC software 21, txCdsPredict software, CNCI software 22, and the pfam database 23. Candidate mRNAs were defined as transcripts with a CPC-threshold greater than or equal to zero, a CNCI-threshold greater than or equal to zero, a txCdsPredict-threshold greater than or equal to 500, or transcripts aligned in; other transcripts were categorized as lncRNAs. Clean reads were aligned using Bowtie2 to the chicken genome (Gallus gallus 4.0, April 2013, Ensembl Build 85) 24. The FPKM was calculated using RSEM software 25. Genes and lncRNAs with a fold change greater than or equal to 2.00 and a false discovery rate (FDR) less than or equal to 0.001 were identified by DEGseq software as differentially expressed (DE) genes or lncRNAs^26^, ^27^. Additionally, GO Ontology was used to determine the distribution of gene function, and KEGG was used to annotate the pathway of the genes with an FDR less than or equal to 0.01.

### iTRAQ procedures

The exosome protein digestion procedure was performed as previously described, with minor modifications 28, 29. The solution was placed into a tissue lyser for 2 min at 50 Hz to release proteins. Following centrifugation at 25,000 x *g* for 20 min at 4°C, the supernatant was transferred into a new tube, reduced with 10 mM DTT for 1 h at 56 ºC, and alkylated for 45 min at 25 ºC in the dark with 55 mM iodoacetamide (IAM). Following centrifugation (25,000 x *g*, 4°C, 20 min), the protein concentration of the supernatant was quantified by Bradford assay and SDS-PAGE. The results of the iTRAQ experiment were analyzed by the Shenzhen Institute of Gene Research (BGI, China). Trypsin Gold (Promega, Madison, WI, USA) in a ratio of 40:1 protein-to-trypsin was used to digest the proteins at 37°C overnight. After digestion, the peptides were desalted with a Strata X C18 column (Phenomenex) and vacuum-dried according to the manufacturer's protocol. The peptides were dissolved in 30 μl 0.5 M TEAB with vortexing. The iTRAQ labelling reagents from the iTRAQ Reagent 8-plex kit were brought to RT and combined with the proper peptide samples according to the , short for Isobaric tag for relative and absolute quantitation, is an isobaric labeling method used in quantitative proteomics by tandem mass spectrometry to determine the amount of proteins from different sources in a single experiment.It uses stable isotope labeled molecules that can be covalent bonded to the N-terminus and side chain amines of proteins.manufacturer's protocol. The peptides were separated on a Shimadzu LC-20AB HPLC Pump system coupled with a high pH reversed-phase chromatography (RPC) column and then reconstituted to 2 mL with buffer A (5% acetonitrile (can) and 95% H_2_O). The eluted peptides were pooled and vacuum-dried. Each fraction was resuspended in buffer A (2% acetonitrile and 0.1% formic acid in water) and centrifuged at 20,000 x *g* for 10 min. The supernatant was loaded onto a C18 trap column with a drip rate of 5 μL/min for 8 min using the autosampler function of a LC-20AD nano-HPLC instrument (Shimadzu, Kyoto, Japan). Data acquisition was performed by a TripleTOF 5600 System (SCIEX, Framingham, MA, USA) equipped with a Nanospray III source (SCIEX, Framingham, MA, USA), a pulled quartz tip emitter (New Objectives, Woburn, MA), and the Analyst 1.6 software package (AB SCIEX, Concord, ON). The raw MS/MS data was converted into MGF format using the ProteoWizard tool msConvert, and the exported MGF files were compared to selected databases using Mascot version 2.3.02. IQuant was used to quantitatively analyze the labeled peptides with isobaric tags 30. To assess the confidence intervals of the peptides, the peptide-spectrum matches (PSMs) were pre-filtered at a PSM-level FDR of 1%. In order to control the rate of false-positives identified at the protein level, a protein FDR of 1% was estimated after protein inference based on the Picked protein FDR strategy 31. A protein was determined to be significantly differentially expressed if the P-value was less than 0.05.

### Quantitative real-time PCR

Quantitative real-time PCR (qRT-PCR) was performed using the SYBR Premix Ex Taq ll kit (Takara, Japan) and a BioRAD real-time PCR instrument following cDNA synthesis with the PrimeScript RT reagent kit and gDNA Eraser (Takara, Japan). Primer sets (Table 1) were designed based on differently expressed mRNA sequences downloaded from NCBI (http://www.ncbi.nlm.nih.gov) using an online tool (http://www.ncbi.nlm.nih.gov/tools/primer-blast/) and subsequently synthesized by Sangon Biotech (Shanghai, China). *GAPDH* was used as the internal reference gene and all assays were run in triplicate.

### Analysis of the PRM-MS results

PRM-MS is a type of MS technology used to determine the reliability of iTRAQ MS readings of the immunity protein BPI which as a quality control. PRM-MS was carried out at Shanghai Bioprofile Co., Ltd. (Shanghai, China). Signature peptides for the target proteins were defined according to iTRAQ data. Sixty μg of each protein were prepared, reduced, alkylated, and digested with trypsin, following the protocol for iTRAQ analysis. The peptide mixtures were introduced into the mass spectrometer via a C18 trap column (0.10 × 20 mm; 5 μm), followed by a C18 column (0.75 × 150 mm; 3 μm). The measurements were acquired using a Q-Exactive mass spectrometer (Thermo Scientific). The acquisition method combined scan events corresponding to a full MS scan and a PRM scan with inclusion list. The instrument settings for the MS scans were as follows: resolution at 70,000@m/z 200, scan range from 350 to 1800 m/z, auto gain control (AGC) target set to 3e6, and the maximum injection time set at 2000 ms. The PRM method involved a resolution of 35,000@m/z 200, an AGC target of 3e6, a maximum IT of 150 ms, HCD MS2 activation, an isolation window of 2.0 Th, and a normalized collision energy of 30. Data analysis was performed using Skyline version 4.1. Statistical significance determined by student's T-test, P<0.05.

### Statistical analysis

Statistical analyses and some graphical representations were performed using GraphPad Prism 5 software. P<0.05 was considered statistically significant. The specific statistical tests used in each experiment are indicated in the corresponding figure legend with means +/- standard deviations (SD).

## Results

### Analysis of the differential expression of mRNAs and lncRNAs in the exosomes of ALV-J infected HD11 cells

**Fig 1:**
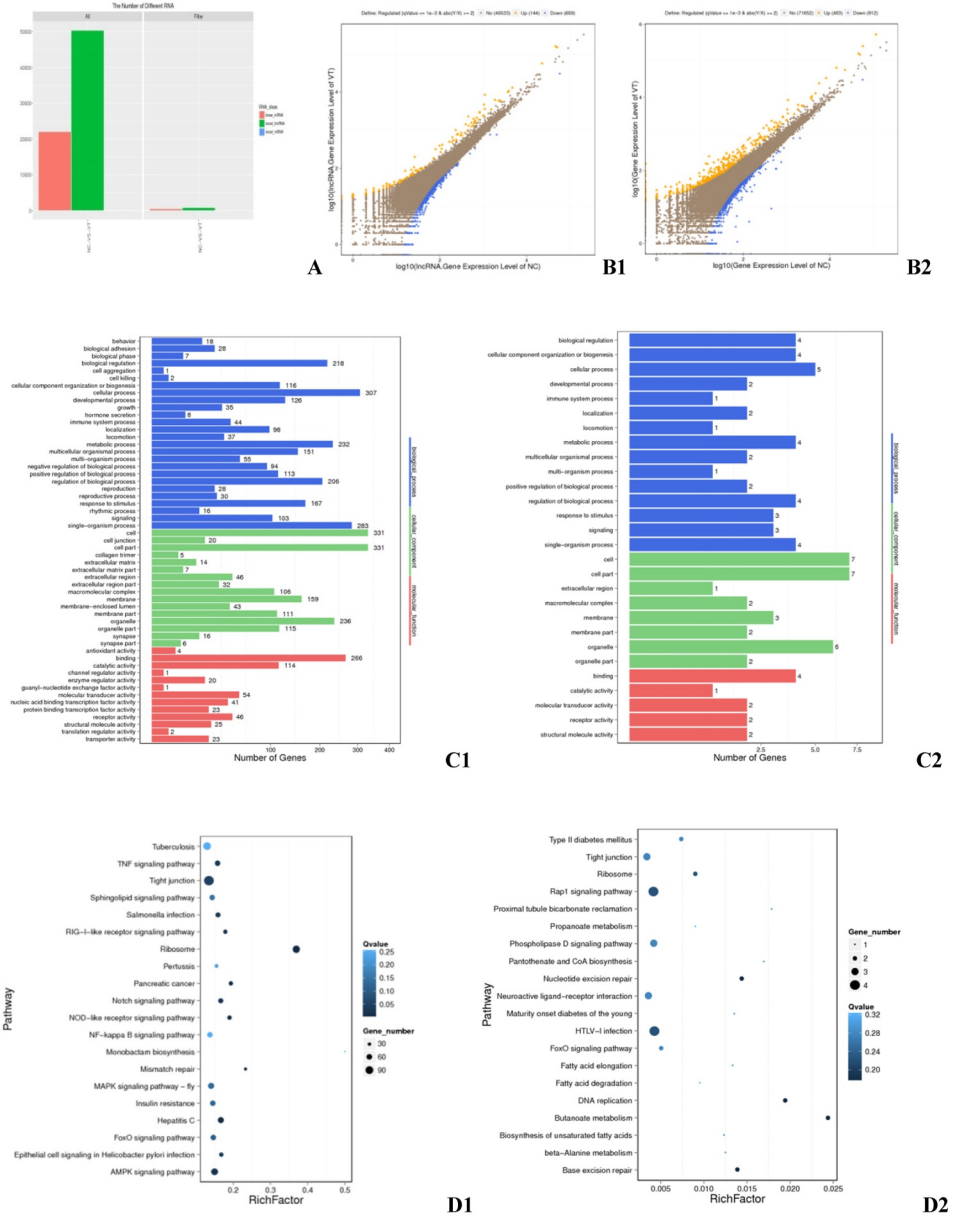
** Differentially expressed genes in the exosomes of infected and control HD11 cells. (A) All mRNAs and lncRNAs DE between the negative control (NC) and virus-injected (VT) groups.** X axis: Difference scheme, Y axis: Numbers of RNAs, Colors: Classification of RNAs. **(B) Differential expression of genes between the NC and VT groups.** B1: mRNA, B2: lncRNA. X axis: compare the gene expression quantity of a certain sample with logarithm of the expression quantity; Y axis: compare the gene expression quantity of another copy in the scheme, and take logarithm of the expression quantity; Colors: blue indicates down-regulation, orange indicates up-regulation, and brown indicates non-significant difference. **(C) GO annotation of DEGs between the NC and VT groups.** C1: DEGs, C2: differential target genes. X axis: Number of genes, Y axis: GO item, color: GO category. **(D) KEGG pathway analysis of DEGs between the NC and VT groups.** D1: DEGs, D2: differential target genes. X axis: enrichment factor, Y axis: pathway, Color: p value size, Size: number of genes.

RNA-seq detected a total of 93,929 transcripts, 56,548 of which were lncRNAs and 37,354 of which were mRNAs (Fig. 1A). Genes and lncRNAs with a fold change greater than or equal to 2.00 and a FDR less than or equal to 0.001 were determined to be differentially expressed (DE). RNA-sequencing identified 513 DEGs, 310 of which were up-regulated in ALV-J infected cells and 203 of which were down-regulated (Fig. 1 B1). Gene ontology (GO) and Kyoto Encyclopedia of Genes and Genomes (KEGG) analysis revealed that these DEGs are involved in the TNF signaling pathway, the formation of tight junctions, ribosomal function, the AMPK signaling pathway (Fig. 1 D1), which important to the biological functions such as cellular process, cell part, organelle part, and catalytic activity (Fig. 1 C1). Ontology also revealed that a striking 44 of the DEGs identified were involved in immune system processes (Fig. 1 C1). A total of 843 DE lncRNAs were found, among which 144 were up-regulated and 699 down-regulated (Fig. 1 B2). These DE lncRNAs had target genes in the pathways of Rap1 signaling, tight junction formation, HTLV-I infection (Fig. 1 D2), as well as targets involved in key cellular processes, cell part, organelle, and molecular transducer activity (Fig. 1 C2). One of the target genes was found to be involved in immune response (Fig. 1 C2).

### Analysis of mRNAs annotation

**Fig 2:**
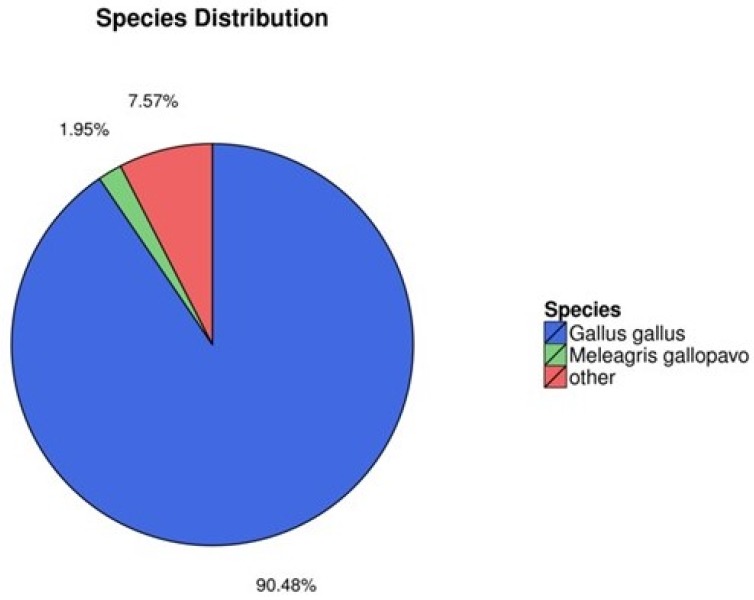
Species annotation of the identified mRNAs (colors: species).

In order to better understand gene function, we annotated the assembled new mRNA and the known mRNA. Blast 32 and Diamond 33 were used to annotate mRNA with the non-redundant nucleotide sequences (NT), non-redundant protein database (NR), clusters of orthologous groups (COG), KEGG, and SwissProt databases, and the Blast2GO software 34 was used to annotate the result of NR annotation. The results of annotation are summarized in Fig. 2. Of all mRNAs surveyed, 90.48% were annotated with Gallus gallus, while 7.57% were annotated with other species (Fig. 2). These mRNAs—such as the *ENV polyprotein* (coat polyprotein), *avian retrovirus envelope protein, gag gene protein p24* and *retroviral gag p10 protein*—were related to ALV.

### Analysis of the differential expression of proteins in the exosomes of ALV-J infected HD11 cells

iTRAQ analysis generated 278,905 spectra, with 791 peptides and 388 proteins identified within 1% FDR. Table 2 briefly summarizes the data collected from the iTRAQ analysis.

**Fig 3:**
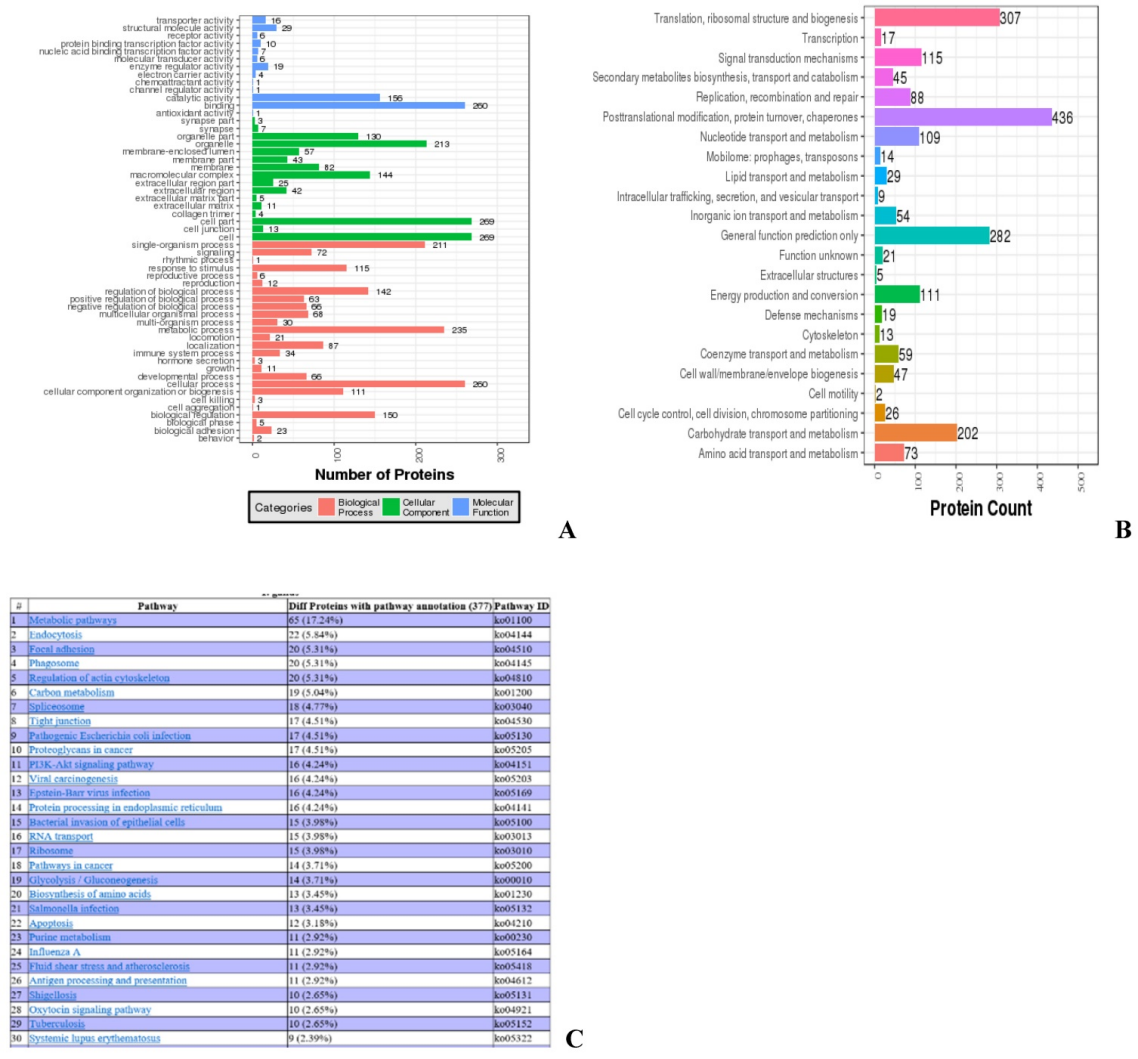
** Ontology analyses for all identified proteins. (A) Gene ontology analysis of the proteins.** Colors: Different GO categories. **(B) COG analysis of the proteins.** X axis: COG items, Y axis: number of proteins. **(C) Web result of the pathway analysis of the proteins.**

Most of the identified proteins are involved in biological functions, such as organelle, catalytic activity, and metabolic processes, while 34 proteins are involved in immune response (Fig. 3A). COGs of proteins were delineated by comparing protein sequences encoded in complete genomes, representing major phylogenetic lineages. Most proteins analyzed this way are involved in biological functions, such as posttranslational modification, protein turnover, chaperone activity, translation, and ribosomal structure and biogenesis, while 19 proteins are involved in cellular defense mechanisms (Fig. 3B). KEGG analysis was used to determine the top 30 pathways in which the identified proteins are involved. The top three different proteins with pathway annotation are: 65 proteins (17.24%) are involved in metabolic pathways, 22 proteins (5.84%) are involved in endocytosis, and 20 proteins (6.31%) are involved in focal adhesion. Some of the proteins identified are involved in immunity; 13 proteins (3.45%) are involved in salmonella infection, seven proteins (1.86%) are involved in HTLV-I infection, and four proteins (1.06%) are involved in the toll-like receptor signaling pathway (data not shown) (Fig. 3C).

DEPs were defined as those with a 1.2 fold change (mean value of all comparison groups) between the NC and VT groups and a P-value less than 0.05 (t-test of all comparison groups). A total of 50 DEPs were identified, 25 of which were up-regulated and 25 of which were down-regulated.

**Fig 4:**
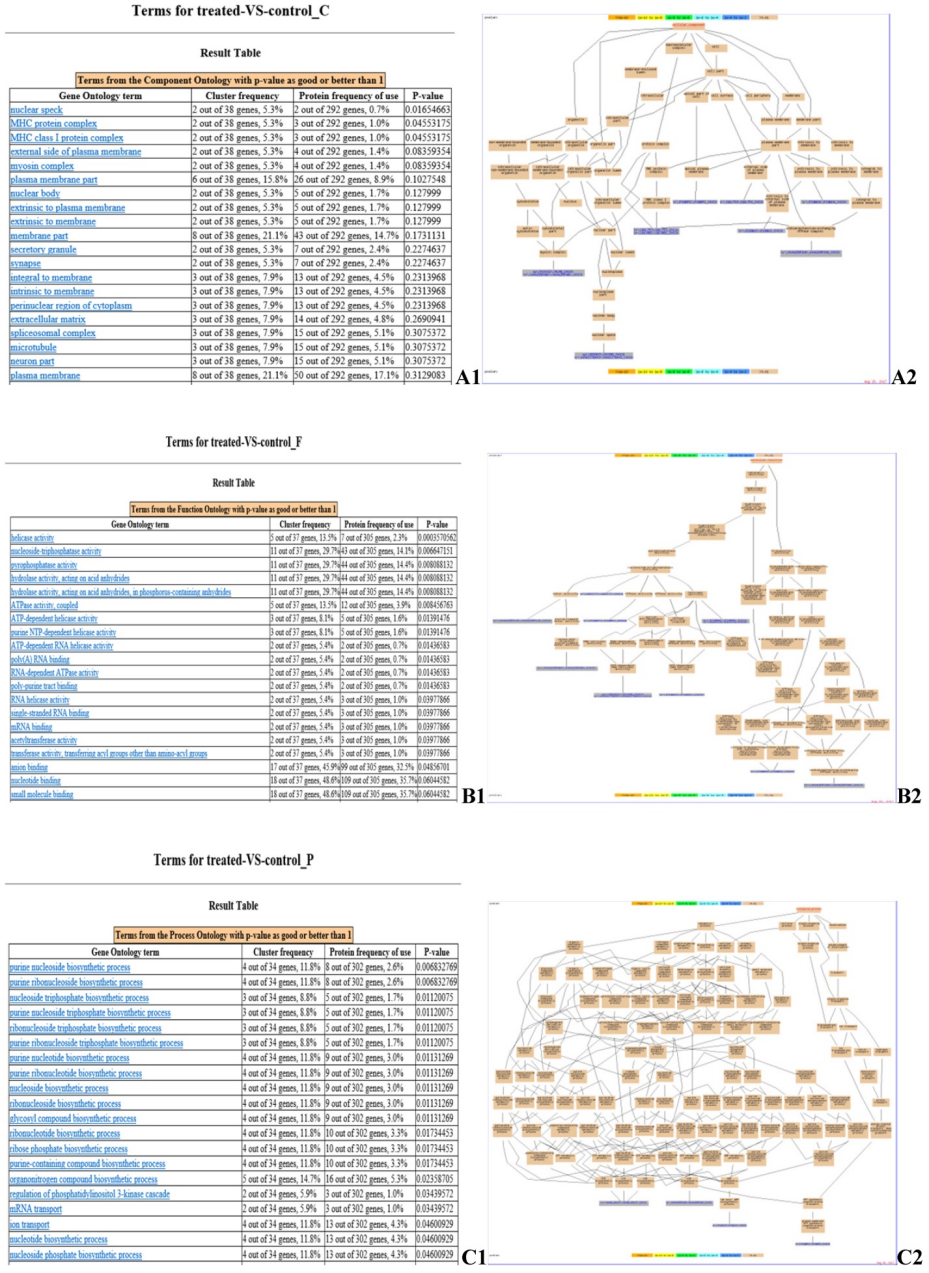
** GO enrichment analysis of identified DEPs.** Cluster frequency indicates the ratio of annotation of the same GO term between all DEPs and all proteins identified. **(A) Gene ontology analysis of the proteins in cellular component.** A1: GO enrichment results screenshot, p-value <0.05 is a significantly enriched GO entry; A2: Genes annotated to the term.** (B) Gene ontology analysis of the proteins in molecular function.** B1: GO enrichment results screenshot, p-value <0.05 is a significantly enriched GO entry; B2: Genes annotated to the term. **(C) Gene ontology analysis of the proteins in biological process.** C1: GO enrichment results screenshot, p-value <0.05 is a significantly enriched GO entry; C2: Genes annotated to the term.

GO enrichment analysis identified the pathways in which the DEPs are involved (Fig. 4). Myosin regulatory light chain 2, MY05A, DDX39B, EIF4A3, BFI, beta-2-microglobulin, ATP6V1A, SERPINE2, ATPase subunit alpha-3-like, and PLG (Fig. 4 A2) are located in cellular compartments, such as the nuclear speck, are involved in the formation of MHC protein complex, MHC class I protein complex, and myosin complex (Fig. 4 A1). DCTN2, MY05A, PSMC3, EIF2S3L, DDX39B, EIF4A3, MCM6, MOV10, UPF1, and ATP6V1A (Fig. 4 B2) are involved in molecular functions, such as helicase activity, nucleoside-triphosphatase activity, pyrophosphatase activity and ATP binding (Fig. 4 B1). NME2, ATPase subunit alpha-3-like, CATHL1 and ATP6V1A (Fig. 4 C2) are involved in biological processes, such as the purine nucleoside biosynthetic process, transport, immune response and primary metabolic processes (Fig. 4 C1).

**Fig 5:**
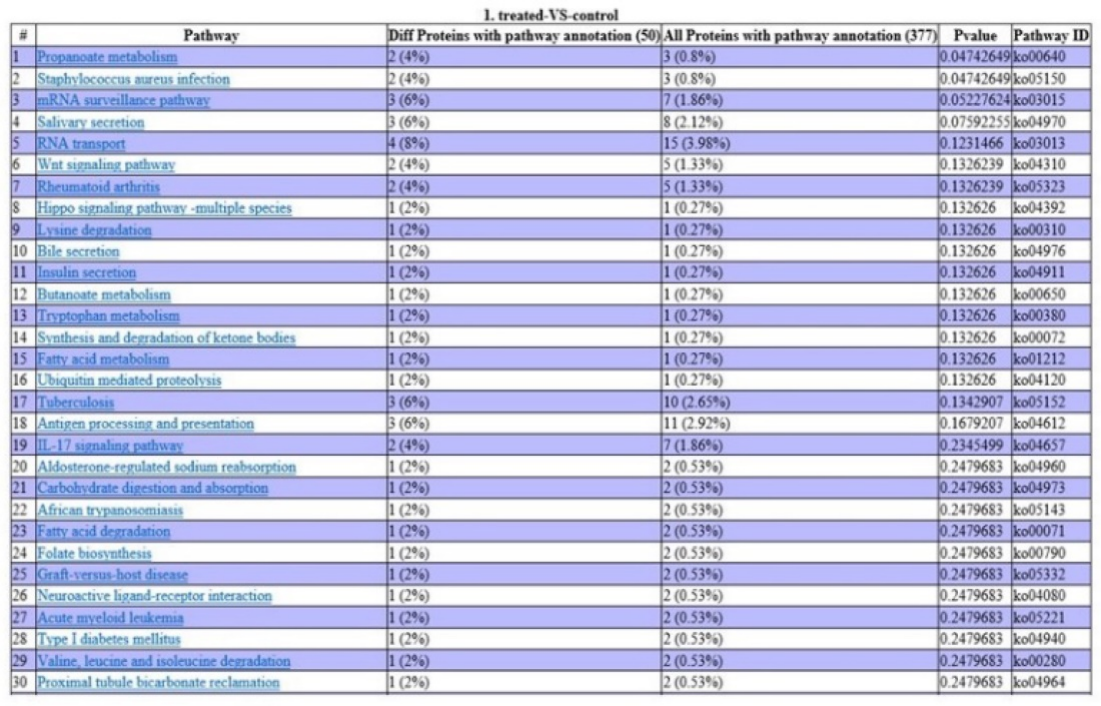
** Pathway enrichment analysis of DEPs.** P-values indicate the level of protein enrichment for each pathway term.

Analysis using the KEGG database revealed that two DEPs are involved in propanoate metabolism, two in staphylococcus aureus infection, two in the IL-17 signaling pathway, three in antigen processing and presentation, three in tuberculosis infection, and one in acute myeloid leukemia (Fig. 5).

### Joint analysis of the transcriptome and the proteome

Joint analysis of the transcriptome and proteome revealed correlations between DEGs and DEPs that would not have been seen with individual set analysis only. The data screening and difference definition of the correlation analysis is summarized in Table 3.

**Fig 6:**
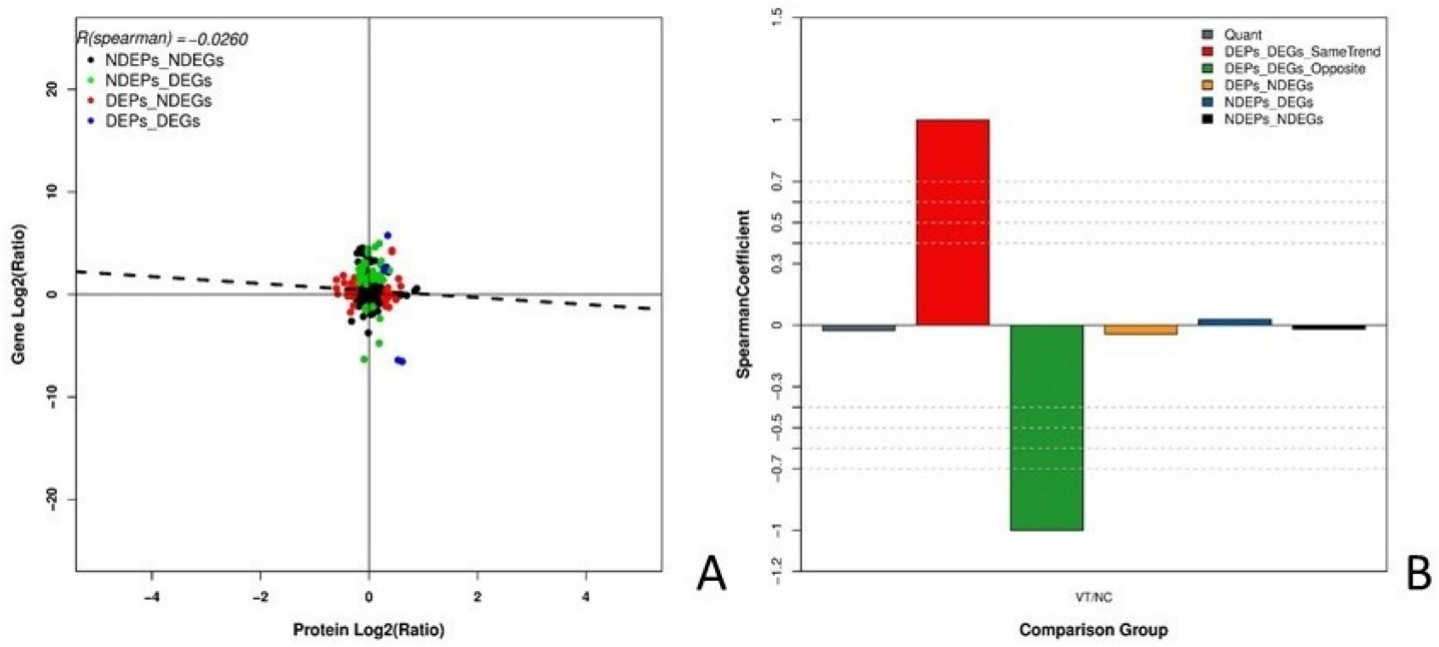
** Joint analysis of the DEGs and DEPs in NC or VT HD11 cells. (A) The correlation of all quantitative proteins and genes.** The abscissa represents protein expression levels and the ordinate represents gene expression levels. Black dots indicate that both the mRNAs and proteins have no significant differences; red dots indicate that there is no significant difference in the mRNAs, but the proteins have significant differences; green dots indicate that mRNAs have significant differences, but proteins do not have significant differences; blue dots indicate that both the mRNAs and proteins have significant differences. **(B) The correlation coefficient of quantitative results.** The abscissa represents the comparison group and the ordinate represents the spearman coefficient. Six types correlation coefficients were estimated: 1) all quantitative protein and mRNA associations, 2) proteins and mRNA with the same trend of significant difference, 3) proteins and mRNAs with the opposite trend of significant difference, 4) proteins that are significantly different but mRNAs without significant difference, 5) mRNAs that are significantly different but proteins without significant difference, and 6) proteins and mRNAs without significant differences.

Joint analysis identified 50 DEPs and 7015 DEGs between the NC and VT groups. This analysis also revealed that there are five instances in which both the gene and protein differential expression patterns are positive. The quantification, significant differences, and quantity of these categories are summarized in Table 4. Table 5 was listed the five correlations, they were HBAD, SERPINE2, CATHL2, ADSS and LDHB. The correlation between the expression levels of mRNA and protein in each group was analyzed (Fig. 6 A), and the R (spearman) value was found to be -0.0260. The correlation coefficient of the following comparison groups was determined: 1) DEPs and DEGs with the same trend, 2) DEPs and DEGs with the opposite trend, 3) DEPs and non-differentially expressed genes (NDEGs), 4) non-differentially expressed proteins (NDEPs) and DEGs, and 5) NDEPs and NDEGS (Fig. 6 B). The R (spearman) value for each of these categories is 1.0000, -1.0000, -0.0430, 0.0279, and -0.0196, respectively.

**Fig 7:**
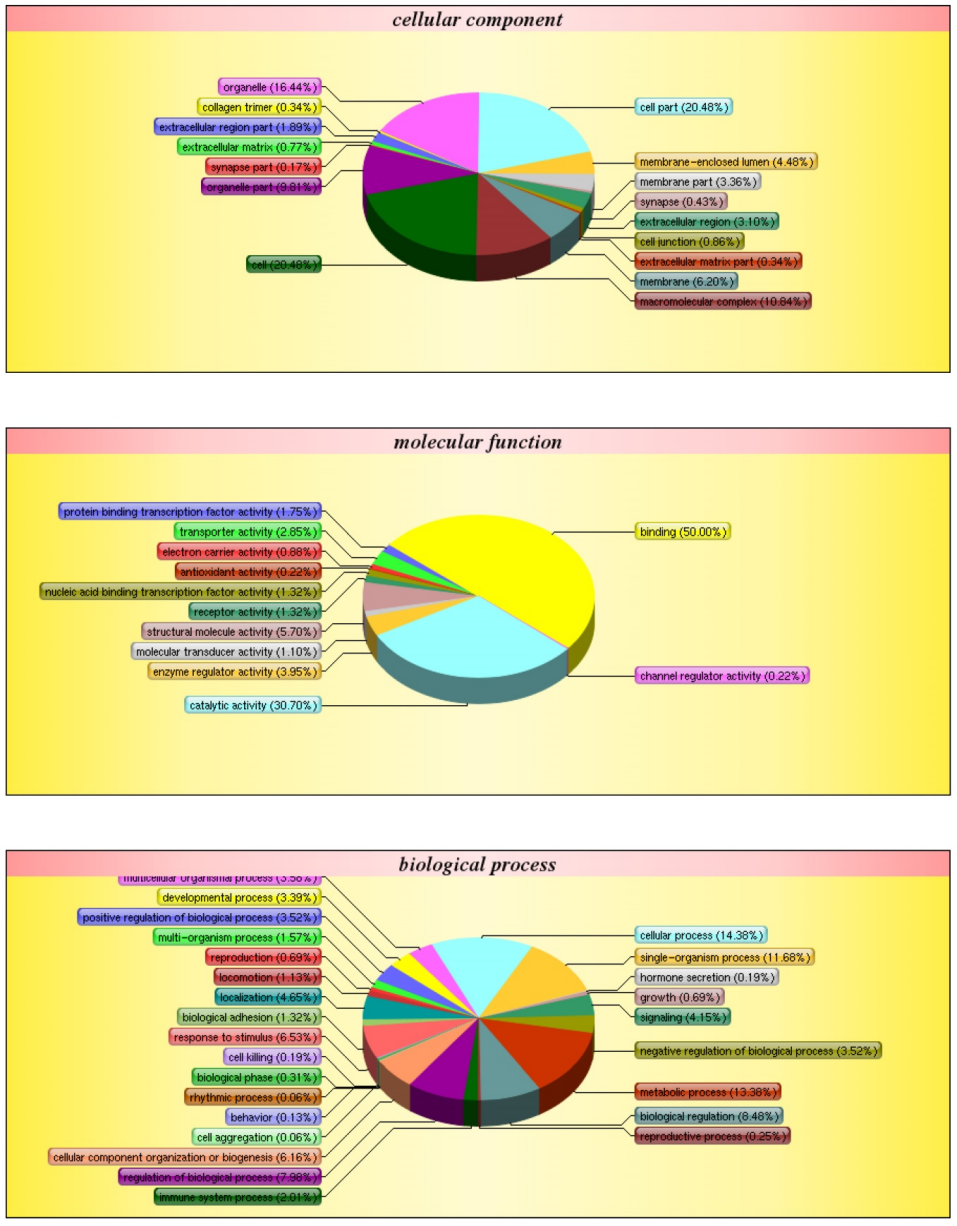
** GO classification**. The diagram shows the distribution of each target in the three ontologies. The different colors are marked as the various items involved in the three ontology. The pie chart represents the percentage of items in the total protein pool.

After obtaining genetic structure and sequence information, GO annotation was carried out on all correlated genes and proteins to determine functional information (Fig. 7). Of all the genes examined, 14.38% are involved in cellular processes, 11.68% in single-organism processes, 13.38% in metabolic processes, and 2.01% in immune response. Additionally, 16.44% of the genes are involved in organelle-related processes, 20.48% in cell part, and 20.48% in cell. Finally, 50.00% of genes are involved in binding, 30.70% in catalytic activity, and 3.95% in the regulation of enzymatic activity.

**Fig 8:**
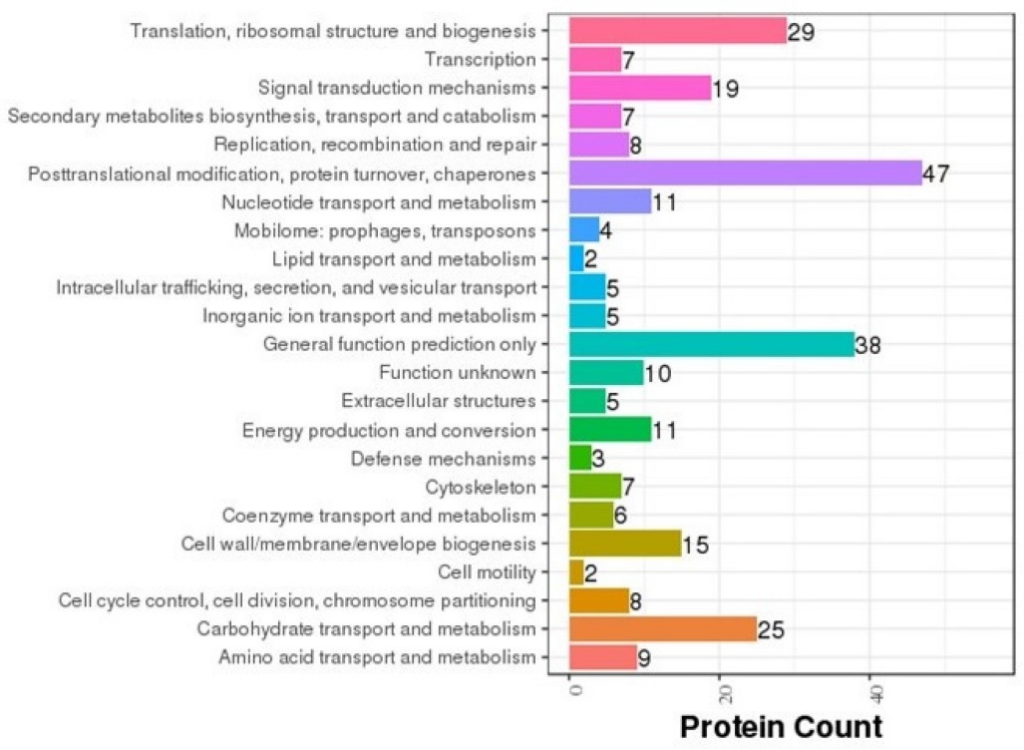
** COG classification**. The abscissa represents protein count and the ordinate represents the COG classification entry.

**Fig 9:**
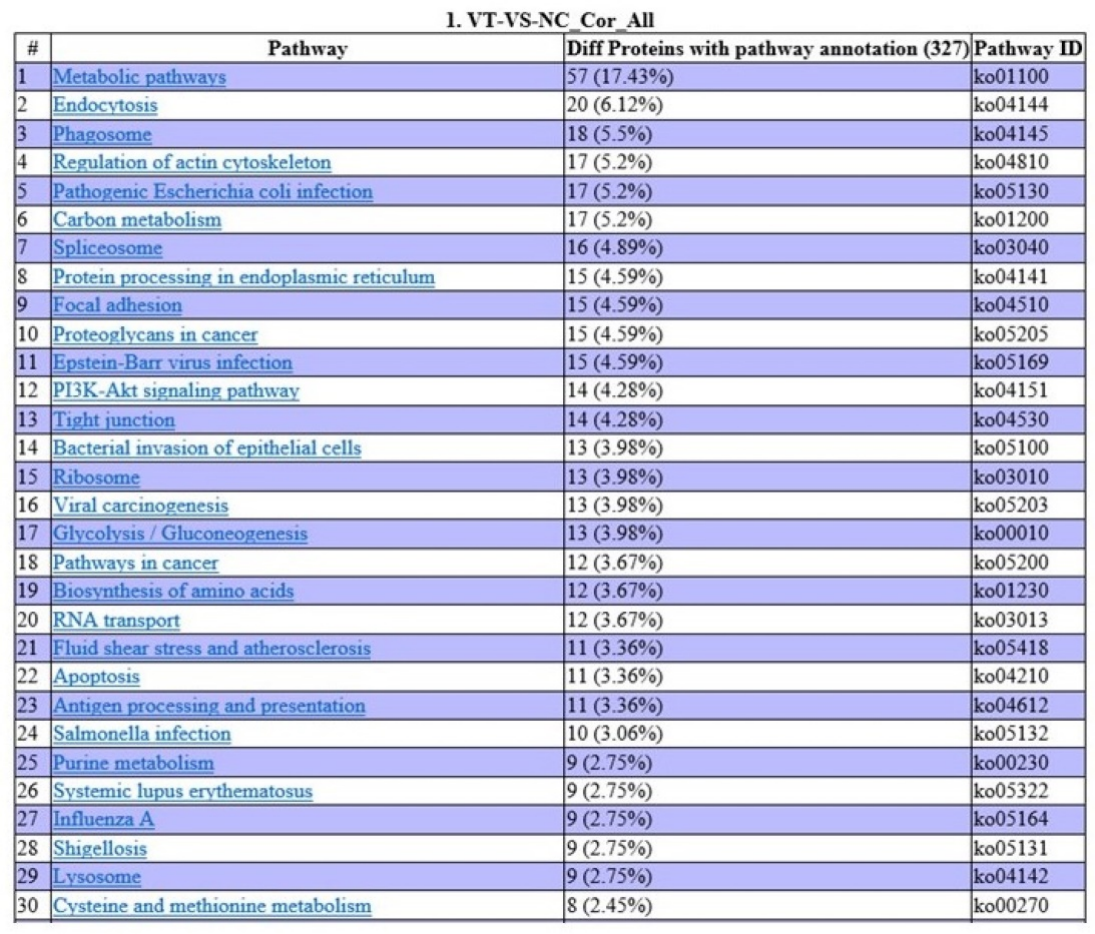
Pathway enrichment analysis of all correlated proteins and genes.

COG annotation was carried out on various proteins based on the results of the correlation and classification of the proteins identified (Fig. 8). Of the proteins analyzed, 47 are involved in posttranslational modification, 38 in general cellular functions, 29 in ribosomal structure and biogenesis, 25 in carbohydrate transport and metabolism, and three in cellular defense mechanisms. In order to determine the main biochemical and signal transduction pathways in which the analyzed proteins are involved, pathway enrichment analysis was conducted (Fig. 9). Analysis revealed that 57 proteins are involved in metabolic pathways, 20 in endocytosis, and 18 in phagosomal processes. In relation to immune response, 12 proteins were identified as being involved in cancer pathways, 11 in antigen processing and presentation, and 10 in salmonella infection.

**Fig 10:**
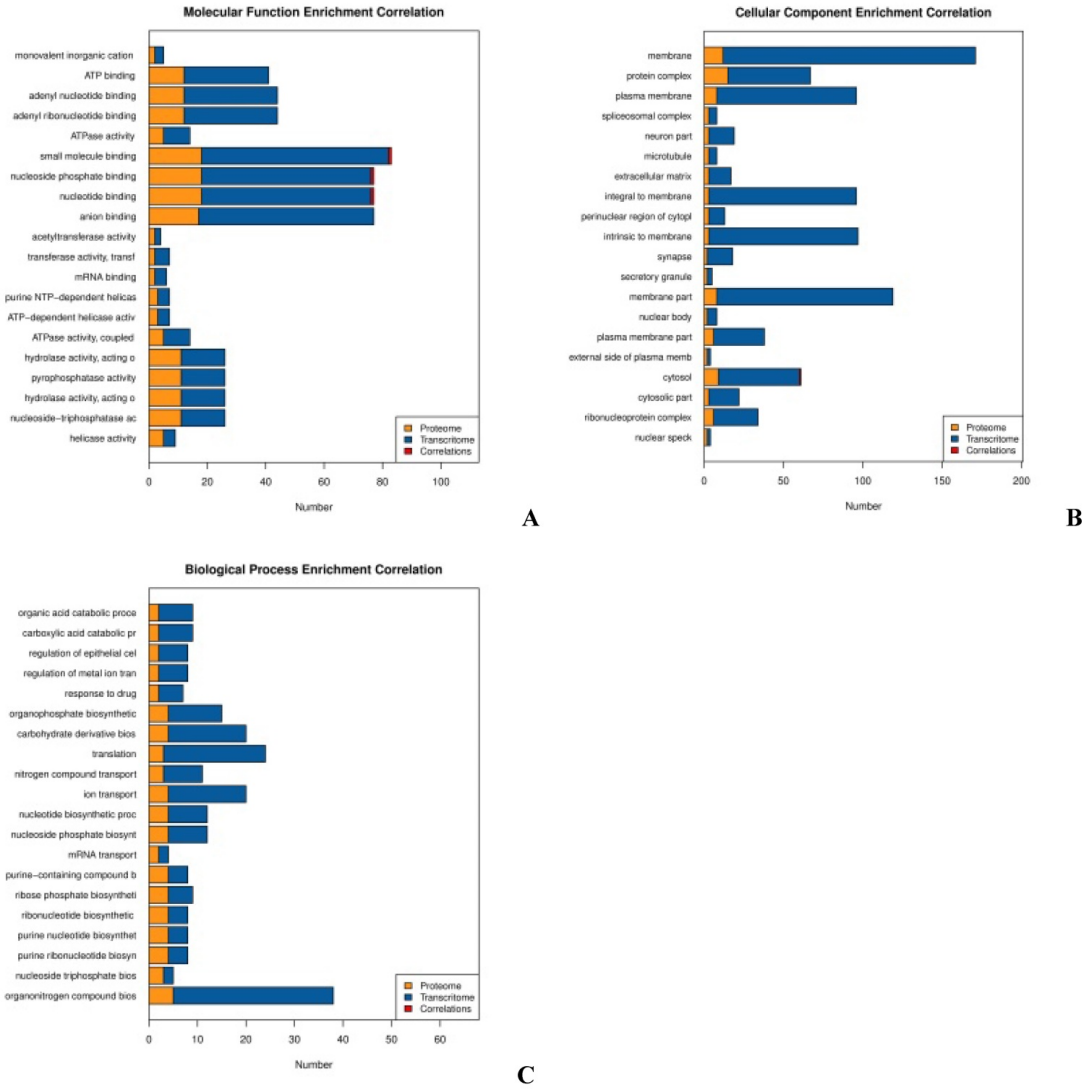
** GO enrichment integration analysis.** Each chart includes protein/gene function, the number of genes or proteins involved, and the number of related statistical graphs. **(A) Molecular function**,** (B) Cellular components**, and** (C) Biological processes**.

DEPs of the proteomic analysis and the DEGs of the transcriptomic analysis were annotated using the GO enrichment integration analysis. GO categories were classified according to the significance of enrichment of the DEPs and the DEGs (Fig. 10). Involved in small molecule binding pathways are 18 proteins and 58 genes, with one positively correlating set. There are also 18 proteins and 58 genes involved in nucleoside phosphate binding, with one positively correlating set. Analysis revealed that 18 proteins and 64 genes are involved in nucleotide binding, with one positive correlating set (Fig. 10 A). Lastly, analysis revealed that there are nine proteins and 51 genes involved in cytosolic processes, one set of which has positively correlation (Fig. 10 B). There were no protein-gene correlations in the top 20 categories, and these proteins and genes involved were mostly related to biosynthetic processes (Fig. 10 C).

**Fig 11:**
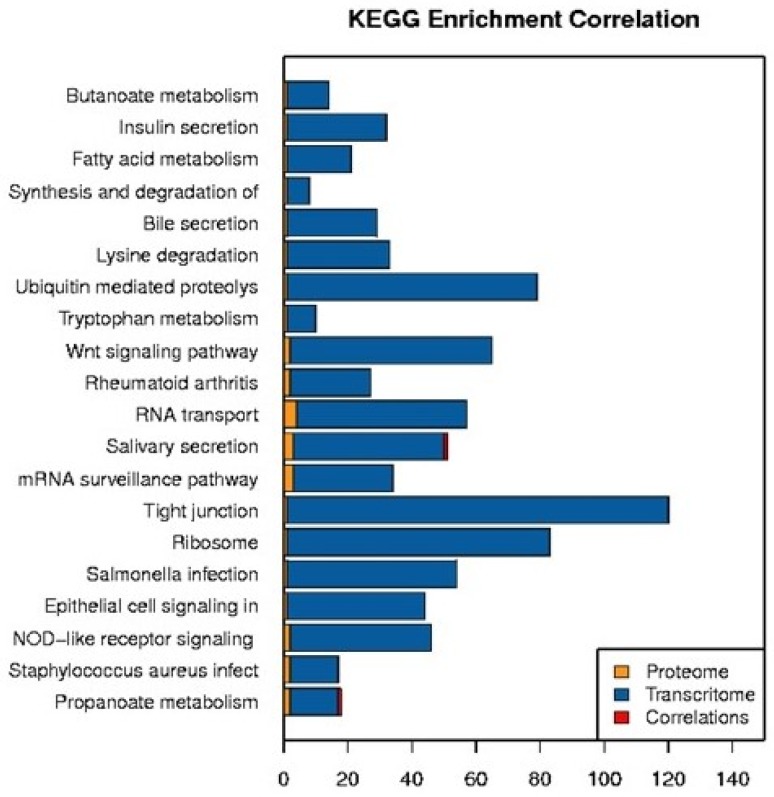
Pathway annotation of the proteome and transcriptome of exosomes isolated from NC or VT HD11 cells.

The transcriptome and proteome were both annotated with metabolic pathways, and the proteins and genes that were noted in the same pathways were correlated and analyzed (Fig. 11). The pathways were classified according to the significance of enrichment of the DEPs and DEGs. In the top 20 categories identified, both the salivary secretion pathway and the propanoate metabolism pathway had one protein-gene positive correlation. The majority of the top 20 categories were related to metabolism. Notably, immune response-related pathways (e.g., the antigen processing and presentation, acute myeloid leukemia, toll-like receptor signaling, leukocyte transendothelial migration, and viral carcinogenesis pathways) were not among the top 20 categories identified.

**Fig 12:**
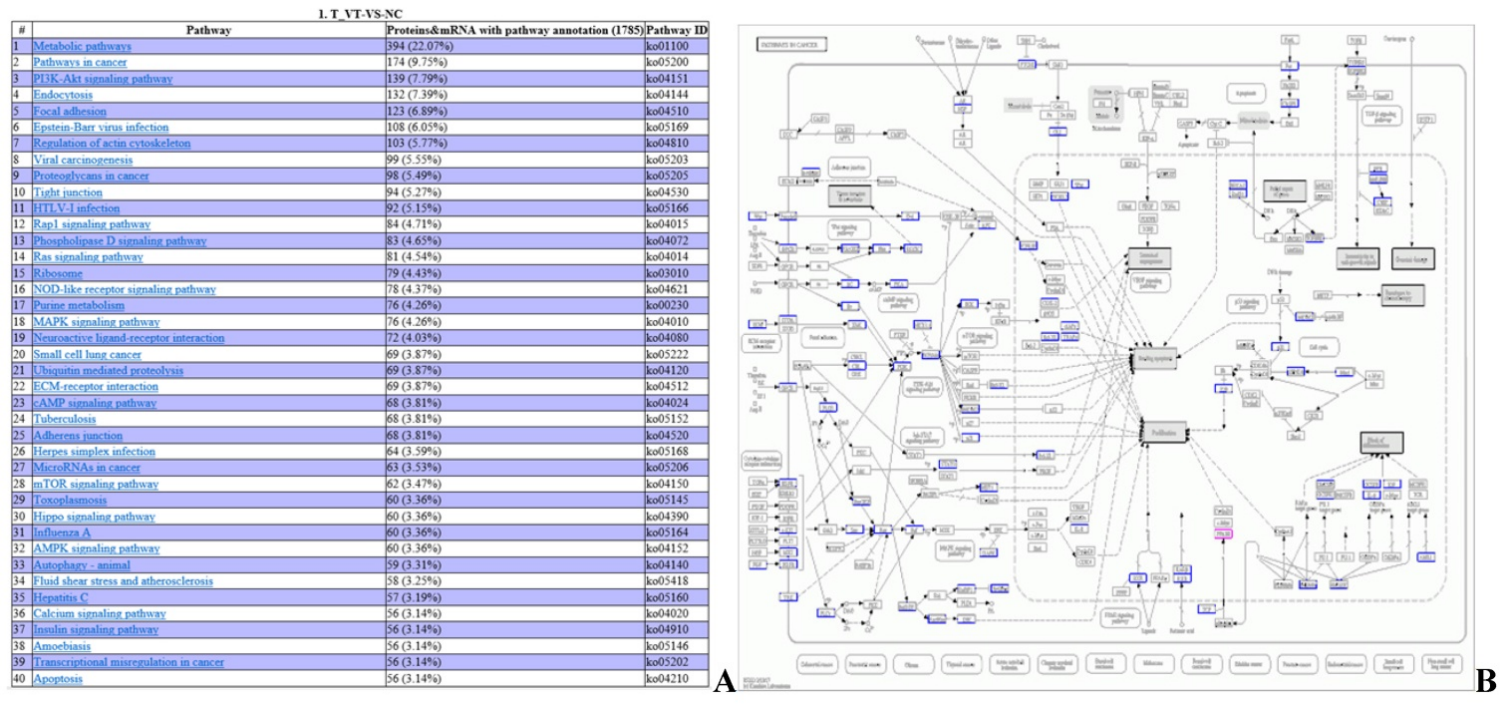
** DEPs and DEGs annotation to KEGG metabolic pathways (A) Integrated analysis of DEP sand DEGs in metabolic pathways. (B) An example for KEGG pathway analysis (pathways in cancer).** The blue box contains the metabolic pathway targets for gene annotation, and the red box contains the metabolic pathway targets for protein annotation.

The DEPs and DEGs obtained from sequencing were annotated using metabolic pathways described in the KEGG database as templates (Fig. 12). DEPs and DEGs annotated to metabolism pathways (394 proteins and mRNAs), cancer pathways (174 proteins and mRNAs), the PI3K-Akt signaling pathway (139 proteins and mRNAs), Epstein-Barr virus infection (108 proteins and mRNAs), viral carcinogenesis (99 proteins and mRNAs), T cell receptor signaling pathway (46 proteins and mRNAs), Th1 and Th2 cell differentiation (43 proteins and mRNAs), and acute myeloid leukemia (24 proteins and mRNAs) pathways (Fig. 12 A). Genes such as ECM, ITGA, FAK, PI3K, and PKB/Akt are involved in cancer pathways which were identified by RNA-seq, and PPARδ was identified by iTRAQ (Fig. 12 B).

### PRM and qRT-PCR analysis of DEPs and DEGs in the NC and VT HD11 cells

**Fig 13:**
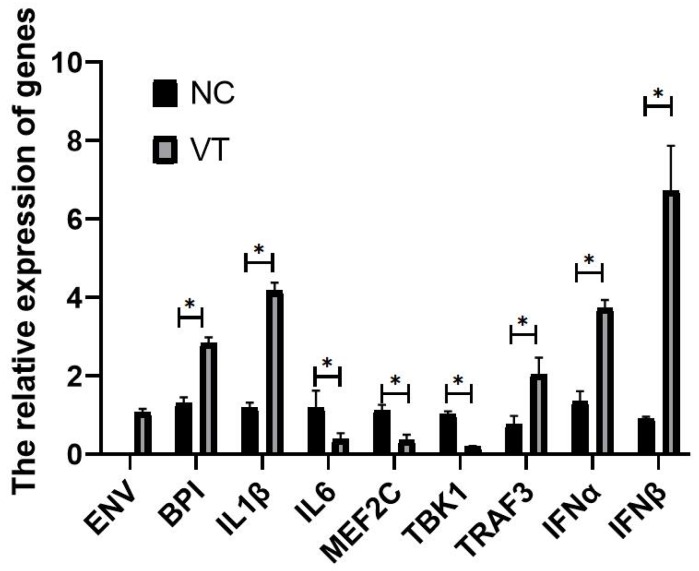
** The relative expression level of genes in exosomes of NC and VT groups.** All values are presented as the means ± SD (n=3). (*) represents statistical significance (P<0.05).

Relative to the *GAPDH* gene, the expression levels of *BPI, IL1β, IL6, TBK1, MEF2C, TRAF3, IFNα* and* IFNβ* mRNA was detected in exosome of NC and VT groups (Fig. 13). Real-time PCR experiments showed that the expression of each gene was consist with the RNA-sequencing results. The Real-time PCR results shown there was no *ENV* detected in NC group.

Experimental analysis revealed that the expression of the target protein BPI was significantly higher in the VT group than that in the NC group. Detailed data analyses, including peptide quantitation, data calibration, and statistical analyses, are listed in Table 6. The difference multiples of these three candidate peptides of protein BPI were around two and with a P-value less than 0.05, indicating that the difference was significant. It means the results of iTRAQ were reliable.

## Discussion

Leukemia can be caused by a multitude of factors, including viral, chemical, environmental, and genetic. For example, avian leukosis is caused by the avian leukosis virus (ALV), which shares many similarities with HIV, including viral protein structure and pathogenic mechanism. Due to these similarities, the study of ALV can greatly contribute to the further understanding of human leukemia and AIDS. Exosomes, which were first identified in the reticulocyte medium, are uniformly sized membranous vesicles secreted by cells in response to stimuli 35. It is thought that exosomes play a vital role in biological development. The study of the role that exosomal secretion plays in the spread of ALV can aid in the understanding of the pathogenic pathways of the virus.

Exosomes originate in the endosome, from which they traffic to the cell membrane for secretion. Exosomes are secreted from most cell types, and they typically contain membrane transporters, fusion proteins (e.g., GTP kinase, Rab family proteins), transmembrane proteins (e.g., CD9, CD63, CD81, CD82), heat stress proteins (e.g., HSP60, HSP70, HSP90), cytoskeletal proteins, actin and tubulin, and lipid-related proteins 36. In fact, CD9 and CD63 often appear in exosomes that they are often used as molecular markers of exosomes in cells 37. In this study, RNA-seq analysis and iTRAQ techniques were used to analyze the transcriptome and proteome, respectively, in the exosomes of HD11 cells infected with ALV-J. The presence of mRNA and peptides corresponding to RAB2A, RAB14, HSP60, HSP70, HSP90, CD9, and CD63 was used to verify the efficacy of exosomal extraction from cells.

Exosomes carry a large number of important nucleic acids, including miRNAs, mRNAs, and lncRNAs. Although most of the mRNA in exosomes is degraded, some of these fragmented mRNAs can be translated into full-length proteins *in vitro* 7, 38. This suggests that the fragmented mRNA carried by exosomes could possibly be translated into full-length proteins after internalization by target cells. Exosomes circulating between cells regulate the biological activity of receptor cells by transporting lipids, proteins, and nucleic acids between them. To activate target cells, exosomes carry and transfer a large variety of cytokines, including fibroblast growth factor (FGF), hepatocyte growth factor (HGF), vascular endothelial growth factor (VEGF), and epidermal growth factor (EGF) 3, 39, 40. Exosomes also play a role in the transfer of EGFR, HGFR, and other receptors, as well as specific active molecules, from receptor cells to target cells through endocytosis 4, 41, 42. We identified key signaling factors (e.g., F2, FN1, ITG, Rac, f-actin, ECM, ITGB, HSP40, HSP70, HSP90, CaM, and PLA) involved in crucial cellular processes, such as the PI3K-AKT signaling pathway, the regulation of the actin cytoskeleton, the RAP1 signaling pathway, the phospholipase D signaling pathway, the RAS signaling pathway, the NOD-like receptor signaling pathway, and the MAPK signaling pathway. In addition, we have identified factors involved in several metabolic-related signaling pathways, such as purine metabolism, ubiquitin processing, proteolysis, and pyrimidine metabolism. We have also found that signaling pathways related to cellular structure—such as endocytosis, focal adhesion, tight junction formation, autophagy, and apoptosis—are regulated by such factors as TGF, RTK, PIP5K, GPCR, ECM, ITGA, ITGB, caveolin, RTK, MLC, Claudin, CTSL, RAS, TRAIL, Fas, and Cathepsin. Exosomes have been shown to contain multiple signaling factors essential to the transmission of various intercellular signals responsible for the regulation of cell proliferation, differentiation, and apoptosis.

Studies have shown that exosomes secreted by host cells play an important role in host viral infection. Exosomes play a dual role in the body's immune response, they spread and proliferate the virus 43-45 as well as promote host immunosuppression 46. Studies have found that, in addition to the traditional recognition mechanism, infected cells can secrete exosomes to transfer viral contents to other uninfected cells 47. In 2014, Jaworski et al. found that HTLV-I infected cells secreted exosomes containing HTLV Tax proteins and mRNA, HBZ mRNA, and ENV mRNA 48, demonstrating that exosomes can be essential to viral proliferation. Through exosomes, HIV-1 can stimulate macrophages to produce inflammatory cytokines. Sampey et al. found that the exosomes of HIV-1 infected cells contained high levels of TAR RNA. Additionally, TAR molecules can combine with TLR3/7/8 to activate the NF-kB signaling pathway and cause inflammation in the host cell 49. Research has shown that exosomes secreted by cells infected with ALV activate the immune response of CD4^+^ and CD8^+^ T cells. Strikingly, immune activation decreased with increasing exosomal secretion, which suggest that the exosome may provide an ideal environment for viral proliferation and cause immune suppression in the host 11. The genome of ALV-J contains gag (including the p19, p2, p10, p27, p12, and p25 genes), pol (including the RT and IN genes), and ENV (including the gp85 and gp37 genes) genes. In this study, we identified the pol that is the pre-enzyme of reverse transcriptase and the gag that is the specific antigen of ALV-J in ALV-J infected cells. Although we identified the ENV mRNA key to ALV-J tumorigenesis, we did not identify its protein counterpart in ALV-J infected cells. This suggests that, while exosomes carry ALV-J, the virus is not active until it is transferred into the host cell. In this study, the *CREB, TFIID* and *TORC* which related to the viral expression were down regulated in the exosome of HD11 cells infected with ALV-J. And the *MET, K-Ras* and *AML1-ETO* which related to oncogenes were down regulated while *c-KIT* and *PML-RAR* were up regulated. These genes indicate the exosomes carry the inhibiting signals to the viral gene expression.

Exosomes are vital to the regulation of host immune response and tumor proliferation. Several studies have shown that many cell types can release exosomes, such as B lymphocytes, T lymphocytes, dendritic cells and mast cells 50-52. Exosomes were also related to some antigen presentation reaction processes. Exosomes secreted by antigen presenting cells contain MHC molecules that can activate the immune response. Some T cells are even able to identify and capture the exosomal MHC molecules. For example, both CD4^+^ and CD8^+^ T cells can recognize exosomal MHC molecules, pass the antigen to T cells, and activate the T cells, thereby activating the host immune response 53. Exosomes are also secreted by infected cells. Studies have observed exosomes secreted from macrophages infected with the mycobacterium bovine tuberculosis and endothelial cells infected with cytomegalovirus. These exosomes contained antigens from the relevant pathogen, which can go on to activate virus-specific responses from CD4^+^ and CD8^+^ T cells in the host 54..In this study, the *α5β1, FAK* and *Paxillin* were down regulated and *FN, Src, Actin, Moesin* and *Radixin* were up regulated that these genes were related to tumor cell migration and invasion. The expression of *TBK1* and *CD40* were down regulated and *NFAT, TRAF3, IFNα* and *IFNβ* were up regulated which genes related to T cell stimulation and activation antibody response. The *IL15R* was up regulated may the promote the leukocyte migration. The pro-infalmmatory cytokines *IL-1β* and* IL6* were up regulated. The gene *BHD* was up regulated which might cause the tumor suppressors and the block of differentiation. VEGFR2 were up-regulated which caused the inhibition of tumor angiogenesis. These results shown exosomes not only carry a variety of factors involved in viral infection but also carry factors involved in the transmission of immune signals between cells.

The study of exosomes in avian viruses is still in the beginning stages. In addition to causing tumors, ALV-J also causes immunosuppression and immune tolerance in the host. Immunosuppression is the reduction in the host's ability to fend off viral infection. This is largely because of the apoptosis and necrosis of lymphocytes. Immune tolerance is a phenomenon in which T cells and B cells under the antigenic stimulation cannot be activated and cannot produce specific immune effector cells or antibodies, blunting the host's immune response to infection. Although Wang found the exosomes obtained from the DF1 cells infected by ALV-J enhance CD4^+^T cells and CD8^+^T cells proliferation in liver according the flow cytometry analysis^10^, but there was no further analysis of T cell function. In this stdudy,* NFATc1* and *yc* were up-regulated in the ALV-J infected group, which caused the inhibition of activated Th1 cell. And the *PDCD4* and *p21/Waf-1* were up-regulated which related to growth suppression. The p38 was down regulated and might lead to the prevented Th1 immune response. The E47 which related to self-renewal of T cells was down regulated. In this study, the *cIAPs* and *Casp* were down regulated and the *Bcl-XL, TRAFs, PI3K* and *TRADD* were up regulated, these genes might resistance to apoptosis signal and inhibited premature apoptosis. These results indicate the exosomes carry the factors which may inhibit the T cell thus cause the immune tolerance.

The ALV-J can cause T lymphoblastic leukemia/ lymphoma, B lymphoblastic leukemia/ lymphoma and acute myeloid leukemia 9. In this study, *MLL, AML1, MPO, ETO* and *MEF2C* were down regulated and *N-CoR, PML* and *SMAD1* were up regulated that might cause the difference resistance. The genes *IL6, CD8, CD35, CD41, CD42, CD44* and *CD117* were down regulated and *IL1β, CD116*, *IgM* and *IgD* were up regulated. Abnormal expression of these genes further leads to cell differentiation and proliferation, such as myeloid related dendritic cell, macrophage, mast cell, lymphoid stem cell, double negative cell, B cell, T cell, lymphoid related dendritic cell and NKT cell. These might cause the immunosuppression. These genes suggesting that exosomes play an important role in the development of immunosuppression and immune tolerance in infected host cells. But in this study, these genes were detected by mRNA levels, the protein of genes were no significantly different in ALV-J infected group and negative control group. This indicates the function of exosome as storage vesicles.

## Conclusion

Cells infected with the ALV-J virus secreted exosomes that transmitted signals between host cells. This occurred not only through the presence of proteins such as BPI that transmit the immune signals but also through the presence of viral nucleic acids that directly transmit the virus into other uninfected host cells. Our sequencing results confirmed previous studies on exosomes and further found exosomes may be involved in the immunosuppression and immune tolerance observed in host cells infected with ALV-J. There are still needed more experiments to confirm that.

## Figures and Tables

**Table 1:** Primer sequences of the differentially expressed mRNAs

Gene ID	Types of primer	5'-primers sequence -3'
GAPDH	Forward	AGGACCAGGTTGTCTCCTGT
	Reverse	CCATCAAGTCCACAACACGG
env	Forward	TGCGTGCGTGGTTATTATTTC
	Reverse	AATGGTGAGGTCGCTGACTGT
BPI	Forward	CCACGACCGCATGGTTTACT
	Reverse	CCTTCGGGATCATGGAGTCTGT
IL-1β	Forward	GGATTCTGAGCACACCACAGT
	Reverse	TCTGGTTGATGTCGAAGATGTC
IL-6	Forward	ATCCGGCAGATGGTGATAAA
	Reverse	CCCTCACGGTCTTCTCCAT
MEF2C	Forward	TTTGGGAATGAACAACCGTA
	Reverse	GGAAACCACTGGAGTAGCC
TBK1	Forward	GGTTTGCCAGAATCGGAGT
	Reverse	TGTAAATACTCCTCTGTGCCGT
TRAF3	Forward	GAGGAGTGAGCGAGTGATAGACAGT
	Reverse	AGTCACTCTGTTCTGGAGGGATTC
IFNα	Forward	CAGGATGCCACCTTCTCTCAC
	Reverse	AGGATGGTGTCGTTGAAGGAG
IFNβ	Forward	CCTCAACCAGATCCAGCATTAC
	Reverse	CCCAGGTACAAGCACTGTAGTT

**Table 2:** iTRAQ protein identification overview.

Sample Name	Total Spectra	Spectra	Unique Spectra	Peptides	Unique Peptides	Protein
Gallus	278,905	1,243	1,149	791	757	388

**Table 3:** Main parameters list of the joint correlation analysis between the transcriptome and proteome

Type	Value
**Protein Unique Peptide**	1
**Protein Fold Change**	1.2
**Protein Significant**	*
**Gene Fold Change**	2
**Gene Significant**	<0.001
**GO Significant**	<0.05
**Pathway Significant**	<0.05
**Blast Identity**	100
**Blast E value**	1e-8
**Top number**	20

**Table 4:** The number of correlations between DEGs and DEPs

Group Name	Type	Number of Proteins	Number of Genes	Number of Correlations
VT-VS-NC	Identification	388	87168	337
VT-VS-NC	Quantitative	388	87168	337
VT-VS-NC	Differentially expressed	50	7015	5

**Table 5:** The list of the correlations (“+” means up regulated and “-” means down regualed)

Protein ID	Gene Name	DEPs	Gene ID	DEGs
**tr|A0A1L1RN90|A0A1L1RN90_CHICK**	HBAD	+	NM_001004375.1	-
**tr|E1BWU2|E1BWU2_CHICK**	SERPINE2	+	NM_001083920.1	-
**sp|Q2IAL7|CTHL2_CHICK**	CATHL2	+	NM_001024830.2	+
**tr|F1NS23|F1NS23_CHICK**	ADSS	+	NM_001031521.1	+
**tr|A0A1L1RY84|A0A1L1RY84_CHICK**	LDHB	+	NM_204177.2	+

**Table 6:** Quantitative analysis of target peptides and proteins.

Protein Name	Peptide Sequence	VT-1	VT-2	VT-3	NC-1	NC-2	NC-3	Ratio-VT/NC	P-value	Average Ratio (VT/NC)
**F1NXX9**	VHYEISSLNLR	1.36E+07	1.29E+07	1.22E+07	7.18E+06	5.92E+06	7.92E+06	1.84	0.001145498	1.96
	GEFFSLAHR	9.59E+06	8.55E+06	7.95E+06	4.84E+06	4.56E+06	4.27E+06	1.91	0.001226125	
	LAEGFPLPLPDR	4.46E+07	4.31E+07	4.26E+07	2.21E+07	1.77E+07	2.11E+07	2.14	9.35619E-05	
